# Parental Stress and Mental Health Outcomes Following the October 7th Events: Insights from Israeli Families of Children with Special Needs

**DOI:** 10.3390/bs15020148

**Published:** 2025-01-29

**Authors:** Yitshak Alfasi, Ruth Maytles, Avi Besser

**Affiliations:** 1 Department of Behavioral Sciences, Interdisciplinary Faculty for Science, Health and Society, Hadassah Academic College, Jerusalem 91010, Israel; 2 Department of Social Work, Interdisciplinary Faculty for Society and Community, Hadassah Academic College, Jerusalem 91010, Israel; ruthma@hac.ac.il; 3 Department of Communication Disorders, Interdisciplinary Faculty for Society and Community, Hadassah Academic College, Jerusalem 91010, Israel

**Keywords:** adult attachment patterns, parental sense of competence, perceived social support, intolerance of uncertainty, meaning in life, parental stress, mental health, October 7th attack

## Abstract

The October 7th events precipitated an unprecedented psychological crisis for Israeli families, particularly affecting parents of children with special needs. This empirical study aimed to investigate the psychological factors contributing to parental stress and mental health outcomes in a community sample of 2097 parents, comparing those with children with special needs (*n* = 540) to those with typically developing children (*n* = 1557). Findings revealed that parents of children with special needs exhibited significantly higher levels of attachment anxiety and avoidance, parental stress, and intolerance of uncertainty, while reporting lower levels of parental competence, perceived social support, and mental health. Notably, meaning in life did not significantly differ between the groups. Correlational analyses indicated that attachment anxiety and avoidance were negatively correlated with parental competence, social support, meaning in life, and mental health while positively correlating with parental stress and intolerance of uncertainty. Mediation analyses showed that attachment anxiety and avoidance significantly predicted increased parental stress and reduced mental health, with these effects mediated by lower parental competence and diminished meaning in life. Moreover, intolerance of uncertainty mediated the effect of attachment anxiety on mental health. However, the mediating role of perceived social support on parental stress was absent among parents of children with special needs. These findings underscore the need for targeted interventions that bolster parental resilience, particularly for families of children with special needs during times of crisis.

## 1. Introduction

### 1.1. Background

Parenting, particularly for children with special needs, presents unique and amplified challenges, significantly increasing stress and negatively impacting physical and mental well-being (Wahab & Ramli, 2022). Research consistently shows that parents of children with special needs experience heightened stress and reduced mental well-being, compared to parents of typically developing children. This stems from the added demands of specialized care, financial burdens, and navigating complex healthcare and educational systems (Faramarzi, 2017; Isa et al., 2016; Padhyegurjar et al., 2018). The resulting increased anxiety, depression, feelings of isolation, and burden are well documented, all contributing to poorer mental health (Faramarzi, 2017). The severity and nature of the child’s condition significantly influence this psychological impact, increasing frustration, disappointment, and the risk of depression, thereby further affecting parental stress and mental well-being (Faramarzi, 2017; Wahab & Ramli, 2022). The October 7th, 2023, attacks in Israel provided a unique context for this study, representing a large-scale, highly publicized event that generated widespread fear and uncertainty within the population, creating a significant and immediate stressor, particularly for vulnerable groups, introduce an additional layer of complexity for parents already facing significant challenges.

Existing research in Israel emphasizes the crucial role of socio-emotional factors, such as attachment patterns and perceived social support, in shaping both short- and long-term psychological responses to war and terror, influencing both stress and broader mental health (Ben Avraham et al., 2022; Besser & Neria, 2010, 2012; Besser et al., 2009; Besser & Priel, 2010; Weinberg et al., 2016). Post October 7th attack studies reveal widespread adverse psychological consequences on the general population and college students, affecting both stress and overall mental health (Alfasi & Besser, 2024; Dopelt & Houminer-Klepar, 2024). However, research on the specific impact of these events on parents, particularly those raising children with special needs, and the consequent effects on both their stress levels and overall mental health remains limited. While previous research has explored the challenges faced by parents of children with special needs and the impact of traumatic events on mental health, the combined and interactive effects of these factors, particularly in the immediate aftermath of a major event such as the October 7th attacks, remain understudied. This study addresses this gap by examining the influence of adult attachment patterns and key socio-emotional resources on parental stress and mental health outcomes among Israeli parents, differentiating between those raising children with special needs and those raising children without special needs.

### 1.2. Adult Attachment Patterns and Reaction to Stressful Events

Attachment theory provides a robust framework for understanding the enduring impact of early childhood experiences on response to stressful events throughout life (Bowlby, 1969/1982, 1973, 1980). The attachment behavioral system—an innate, neurobiological program designed to enhance the survival chances of the human species throughout evolution—is activated in times of perceived threat or danger, prompting individuals to seek proximity to close figures. This proximity provides comfort, protection, and support, thereby alleviating fear and tension (Ainsworth et al., 1978/2015). Early life interactions with reliable, sensitive, and responsive caregiving figures foster a sense of a *secure base*, positively influencing emotional and behavioral patterns throughout life and promoting hope, optimism, and effective stress management (Bowlby, 1988).

Building on this foundation, early caregiver-child interactions shape internal working models—mental representations of the self and others—that influence expectations, perceptions, and beliefs regarding the availability and responsiveness of attachment figures in times of need. These models shape behavior in close relationships and coping strategies in emotionally challenging situations (Mikulincer & Shaver, 2016). Secure attachment fosters adaptive coping mechanisms, including problem-focused (actively addressing the problem’s source) and controlled emotion-focused strategies (managing negative emotions constructively) (Shaver & Mikulincer, 2007).

In contrast, individuals with insecure attachment styles, characterized by high levels of attachment anxiety and/or avoidance, experience increased vulnerability, diminished emotional regulation, and doubts about their ability to cope effectively. High levels of attachment anxiety manifest as heightened vigilance toward potential rejection or threats, while high levels of attachment avoidance are expressed as emotional distance and a reluctance to seek support. Both patterns are associated with a general belief in an unpredictable and potentially unsafe world (Bowlby, 1973; Shaver & Mikulincer, 2007). While early research categorized attachment into three styles (secure, anxious, and avoidant (Ainsworth et al., 1978/2015), current research focuses on two dimensions: attachment *anxiety* and *avoidance* (Bartholomew & Horowitz, 1991; Brennan et al., 1998). Attachment anxiety is characterized by preoccupation with rejection and fear of abandonment, while avoidance reflects distrust in others’ availability and a tendency toward emotional detachment. These insecure patterns often lead to maladaptive coping in stressful situations, which may include excessive rumination of negative thoughts or suppression and avoidance (Shaver & Mikulincer, 2007).

The relevance of attachment theory in understanding responses to stressful life events and crises is consistently demonstrated (Simpson & Rholes, 2012). Global events, such as the COVID-19 pandemic, highlight this, with studies showing that individuals with higher levels of attachment anxiety and avoidance experienced significantly greater psychological distress, including increased stress, anxiety, depression, and PTSD (Post Traumatic Stress Disorder) symptoms (Alfasi, 2025; Chi et al., 2020; Karantzas et al., 2020; Moccia et al., 2020; Overall et al., 2021). These findings underscore the need to consider individual differences in attachment patterns when examining responses to collective stress, as experienced in Israel following the October 7th attacks.

### 1.3. Adult Attachment Patterns and Parental Stress

The intricate relationship between attachment patterns and parental well-being is multifaceted and requires a nuanced understanding. While factors like reduced social support and ineffective emotion regulation play a significant role (Kim et al., 2019), the precise mechanisms linking insecure attachment to heightened parenting stress and reduced overall mental health remain a subject of ongoing investigation. Secure attachment is consistently associated with more adaptive parenting, greater self-efficacy, and reduced stress and improved mental health (Howard, 2010; Johnston & Mash, 1989; Kim et al., 2019), but the impact of insecure attachment is more complex and varies across studies.

Several studies highlight the association between insecure attachment and increased parental stress, although the specific patterns differ. Some research emphasizes the link between avoidant attachment and increased parental stress, particularly following the birth of a first child (Rholes et al., 2006; Vasquez et al., 2002), while others demonstrate a stronger association between attachment anxiety and heightened parenting stress (Collins, 1996; Fraley & Shaver, 1998; Nygren et al., 2012; Saisto et al., 2008). These discrepancies may be due to variations in methodology (assessment timing, sample characteristics, specific measures) and the influence of theoretical models of self and other in interpreting findings (Nygren et al., 2012). Nygren et al. (2012), using a large population-based sample of 8122 Swedish parents of toddlers (97% mothers), found a significant positive correlation between relationship-related anxiety and overall parenting stress, with stronger associations for the subscales of parental incompetence and social isolation. Higher levels of attachment avoidance were also associated with increased social isolation (Nygren et al., 2012). Similarly, Rholes et al. (2006) found links between attachment anxiety and avoidance and parenting stress six months postpartum (Rholes et al., 2006), and Vasquez et al. (2002) showed associations between both attachment anxiety and avoidance and parenting stress, especially regarding work-family conflict (Vasquez et al., 2002). This line of research highlights the complex and often inconsistent pattern regarding the association between attachment patterns and parental stress, underscoring the need for further research examining the mediating factors influencing these associations and their impact on overall mental health. Hence, the current study will investigate various possible mediating factors, including parental sense of competence, perceived social support, intolerance of uncertainty, and sense of meaning in life. We selected those four mediators based on their associations with adult attachment patterns and their established relevance to parental well-being and stress resilience in the face of adversity, as supported by prior research. While other factors undoubtedly contribute to parental stress, these four represent key psychological mechanisms extensively studied and proven influential in stress responses. Furthermore, preliminary analyses revealed significant correlations among these mediators, indicating their interconnectedness and mutual influence. Our mediation models account for these relationships. These factors are explored further in the following section, with significant consideration given to the context of the October 7th attacks and the uncertainty and heightened stress they caused for many families.

### 1.4. Mediating Factors Linking Attachment Patterns and Parental Stress and Mental Health

#### 1.4.1. Parental Sense of Competence

Parental self-efficacy and satisfaction, defined as *parental sense of competence*, are key predictors of stress and well-being among parents (Howard, 2010; Johnston & Mash, 1989). High parental competence buffers against stress, while low parental competence is linked to increased stress, psychological distress, and potentially negative parenting outcomes. Accordingly, a secure attachment pattern plays in important role in fostering higher self-efficacy, reflecting confidence in managing parenting challenges (Johnston & Mash, 1989; Kim et al., 2020). In contrast, insecure attachment is linked to lower self-efficacy and satisfaction among parents (Vogel & Wei, 2005), with mediating factors such as psychological distress and social support. Previous studies have consistently shown that parents of children with special needs often report lower levels of parental competence, highlighting the challenges they face (Fisman et al., 1989; Katkić et al., 2017; Rodrigue et al., 1990). The strength of the emotional bond between parent and child (affective attachment) has been found to positively impact parental competence (Goodman & Glenwick, 2012). Higher levels of self-efficacy and satisfaction are associated with reduced stress and improved mental well-being, while lower levels of parental competence correlate with increased stress and decreased mental well-being, including increased anxiety and depression.

#### 1.4.2. Perceived Social Support

Strong social support networks mitigate stress and promote resilience (Sarason et al., 1993; Shorey et al., 2003), with secure attachment playing a key role by fostering trust and emotional openness, which facilitate the development and maintenance of supportive relationships (Snyder et al., 1997; Vogel & Wei, 2005). This is especially significant for parents of children with special needs, who often require robust social support systems (Shorey et al., 2003). On the other hand, insecure attachment undermines perceived support, increasing stress and distress (Vogel & Wei, 2005). Insecurely attached individuals’ negative perceptions of others hinder support seeking and exacerbate difficulties (Mikulincer & Florian, 1995; Mikulincer et al., 1993). Research on Israeli citizens exposed to missile attacks supports these theoretical assumptions and demonstrates the mediating role of perceived social support in the association between attachment insecurity and PTSD symptoms (Besser & Neria, 2012).

#### 1.4.3. Intolerance of Uncertainty

Intolerance of uncertainty (IU) is defined as a tendency toward negative cognitive, emotional, and behavioral responses to situations characterized by ambiguity and a lack of information concerning future outcomes of the current situation (Carleton et al., 2012, 2007). IU has been previously linked to anxiety, depression, and maladaptive coping (Buhr & Dugas, 2009; Sbrilli et al., 2021). IU is particularly relevant during periods of heightened uncertainty, as experienced during the COVID-19 pandemic and following the October 7th attacks (Davenport et al., 2020). IU’s association with various anxiety disorders underscores its importance to mental well-being (Freeston et al., 1994; Mahoney & McEvoy, 2012; Sternheim et al., 2017; Tolin et al., 2003). Accordingly, secure attachment fosters greater tolerance for ambiguity and adaptive coping with stressful events (Nekic & Mamic, 2019), whereas insecure attachment increases IU and maladaptive coping with stress (Boelen et al., 2014; Clark et al., 2021; Masi et al., 2001; Wright et al., 2017).

#### 1.4.4. Meaning in Life

Finding purpose and meaning in life is important for mitigating distress (Frankl, 1959), especially for parents of children with special needs who face prolonged stress and uncertainty (Bekenkamp et al., 2014). A strong sense of meaning in life buffers against the negative physical and mental health consequences of caregiving stress (Bekenkamp et al., 2014). Research on attachment suggests that secure attachment promotes a sense of meaning in life (Bodner et al., 2014; Shaver & Mikulincer, 2012), while insecure attachment hinders the development of meaning, which in turn contributes to heightened distress (Bodner et al., 2014). Previous studies demonstrated this by showing the protective role of meaning in life in mitigating the negative consequences of attachment insecurity (Chen et al., 2023; Shelton et al., 2020).

### 1.5. The Current Study: Overview and Hypotheses

The literature consistently demonstrates that parents of children with special needs face significant challenges, resulting in heightened stress and psychological distress compared to those raising typically developing children. This vulnerability is exacerbated by insecure attachment, which amplifies vulnerability to stress, particularly during significant life disruptions such as the October 7th attacks. These disruptions, including evacuations, alarms, and the emotional strain on families, pose unique and profound challenges for parents of children with special needs. Accordingly, attachment theory serves as a suitable framework for understanding parental stress and coping strategies in times of high stress caused by external factors. To further understand the psychological mechanisms by which attachment patterns relate to parental stress and overall mental health, mediating factors such as parental sense of competence, perceived social support, intolerance of uncertainty, and a sense of meaning in life play will be investigated. By examining a broad sample of parents, including those of children with and without special needs, the current research seeks to clarify how these factors intersect, particularly in the aftermath of the October 7th attacks and the ongoing conflict.

#### Hypotheses

The following hypotheses will be examined:

**H1.**  *Parents of children with special needs will report significantly higher levels of parental stress, intolerance of uncertainty, and significantly lower levels of parental competence, perceived social support, and mental well-being compared to parents of typically developing children. The study’s aim is to better understand how attachment patterns influence both parental stress and mental health and how this relationship is mediated by key socio-emotional factors.*

**H2.**  *Insecure attachment (both anxious and avoidant) will be negatively correlated with parental competence, perceived social support, mental health, and meaning in life and positively correlated with parental stress and intolerance of uncertainty.*

**H3.**  *Parental competence, perceived social support, and meaning in life will be positively associated with mental health and negatively associated with parental stress. Intolerance of uncertainty will be positively associated with parental stress and negatively associated with mental well-being.*

**H4.**  *The relationships between insecure attachment (anxiety and avoidance) and increased parental stress and reduced mental well-being will be mediated by low parental competence, low meaning in life, low perceived social support, and high intolerance of uncertainty. These mediating variables will, in turn, be associated with increased parental stress and reduced mental health.*

**Figure 1:**
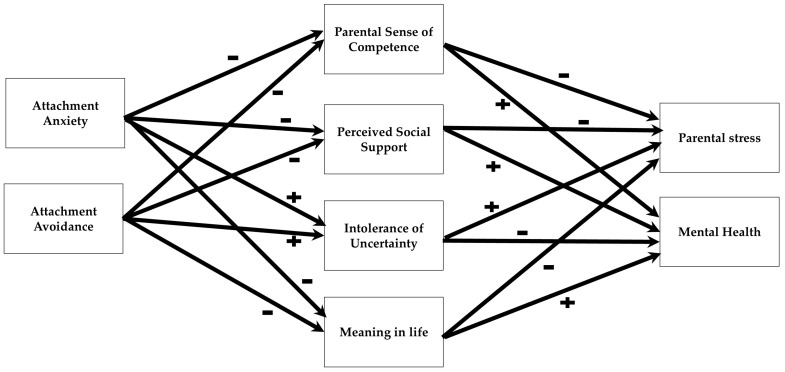
In the aftermath of the October 7th events, this mediational model explores how adult attachment patterns influence parental stress and mental health, proposing that parental competence, social support, tolerance of uncertainty, and the presence of meaning in life act as mediating factors.

Figure 1 presents the proposed mediational model illustrating how adult attachment patterns (anxiety and avoidance) may affect parental stress and mental health following the October 7th attack, with parental competence, social support, intolerance of uncertainty, and meaning in life serving as mediators.

## 2. Materials and Methods

### 2.1. Participants and Procedure

This study utilized a convenience sample of 2097 Jewish-Israeli parents, of whom 540 (25.8%) reported having children with special needs and 1557 (74.2%) reported having children without special needs. The appropriate sample size for this study was determined to be at least 250 based on a power analysis (>0.80) for the average effect size in social-personality psychology (*r* ≈ 0.21; (Richard et al., 2003)) in conjunction with the guidelines for reducing estimation error in social-personality psychology (*n* > 250; (Schönbrodt & Perugini, 2013), but oversampling was deliberately employed in an effort to increase the statistical power of the study. The mean age of children with special needs was 9.56 years (*SD* = 4.52), with 66.6% being boys and 33.4% girls. Diagnoses among these children included physical disabilities (2.9%), learning disabilities (1.7%), attention-deficit disorders (12.4%), participation in special education programs (2.2%), communication disorders (including autism; 5.0%), and emotional disabilities (3.5%). Only participants who identified as parents were included. Recruitment was conducted through *iPanel*, a local online panel of respondents obligated to complete questionnaires. This resulted in a dataset with no missing data. Participants completed a secure online survey, which included obtaining informed consent and completing a demographic questionnaire (capturing gender, age, education, employment, religiosity, marital status, income, place of residence, whether they had a child with a disability, the specific diagnosis, and the child’s age and gender) and measures of adult attachment patterns (anxiety and avoidance), parental stress, mental health, parental sense of competence, perceived social support, intolerance of uncertainty, and meaning in life. This study was not preregistered, but the data file is available on the Open Science Framework (OSF): https://osf.io/u9jyc/ accessed on 20 December 2024.

### 2.2. Questionnaires

#### 2.2.1. Background and Demographics

Participants were asked to provide the following information: gender, age, number of children, and political stance, which was rated on a scale from 1 (*left-wing*) to 7 (*right-wing*). They also reported their cumulative exposure to specific events related to the October 7th attacks through various channels, including the following: exposure to information in the media and on social networks; knowing someone who has been injured, kidnapped, or killed; having a family member who has been harmed in any of these ways; personal involvement in the events of October 7th (as a soldier or participant in the Nova party (https://en.wikipedia.org/wiki/Nova_music_festival_massacre, accessed on 20 December 2024); experiencing an attack on their village; spending time in a security or shelter room; and being evacuated from their home. Additionally, participants provided details about their employment status, religiosity, marital status, education level, monthly gross household income, and place of residence (see Table 1).

#### 2.2.2. The Predictors

Adult Attachment Patterns

Adult attachment patterns were assessed by Wei et al.’s (2007) short form (Wei et al., 2007) of the Experiences in Close Relationship Scale—ECR-S (Brennan et al., 1998), a 12-item self-report questionnaire that measures attachment patterns in adult romantic relationships. The ECR-S demonstrated adequate validity and factor structure (Brennan et al., 1998). While a longer version of the ECR exists (ECR-36), the ECR-S, a shorter, well-validated version (Brennan et al., 1998), was utilized to reduce respondent burden and enhance feasibility in this large-scale study. Previous research has demonstrated that the ECR-S effectively captures the key dimensions of attachment anxiety and avoidance. Participants were instructed to think about their prototype experiences in romantic relationships and rate their agreement with each item on a 7-point Likert scale, ranging from 1 (*strongly disagree*) to 7 (*strongly agree*). In total, six items assessed attachment anxiety (e.g., “I worry that romantic partners won’t care about me as much as I care about them”; α = 0.78), and six items assessed attachment avoidance (e.g., “I try to avoid getting too close to my partner”; α = 0.78).

#### 2.2.3. Mediators

Parental Sense of Competence

Parental sense of competence was assessed using the Parenting Sense of Competence Scale—PSOC (Johnston & Mash, 1989). The PSOC captures both parental efficacy (belief in one’s parenting abilities) and satisfaction (positive feelings toward the parenting role). These two aspects are intertwined; high competence typically leads to greater satisfaction, and vice versa. Accordingly, the scale includes items reflecting both efficacy (e.g., “If anyone can find the answer to what is troubling my child, it is me”) and satisfaction (e.g., “Being a parent is rewarding in itself”). Participants were instructed to respond based on their experiences with their child or, if one of their children was identified as having special needs, to focus on that child when answering. Participants rated their agreement with 17 statements on a 7-point Likert scale ranging from 1 (*strongly disagree*) to 7 (*strongly agree*). Higher scores indicate a greater sense of parenting competence (α = 0.84).

Perceived Social Support

Perceived social support was assessed by the Multidimensional Scale of Perceived Social Support—MSPSS (Zimet et al., 1988), a 12-item self-report measure of social support comprising three subscales, each addressing a distinct source of support: family (e.g., “I get the emotional help and support I need from my family”), friends (e.g., “ My friends really try to help me”), and significant others (e.g., “ There is a special person who is around when I am in need”). The present study used the combined mean score of the three subscales as an index of general perceived social support. Items were rated on a 7-point Likert scale ranging from 1 (*strongly disagree*) to 7 (*strongly agree*), and higher scores indicated higher perceptions of greater perceived social support (α = 0.94).

Intolerance of Uncertainty

Intolerance of uncertainty was assessed using the Intolerance of Uncertainty Scale—IUS-12; (Carleton et al., 2007), which measures responses to uncertainty and ambiguous situations, as well as the extent to which uncertainty is perceived as stressful and disabling (e.g., “Unexpected events upset me greatly” and “Uncertainty prevents me from living a full life”). Items were scored on a 7-point Likert scale ranging from 1 (*strongly disagree*) to 7 (*strongly agree*), with higher scores indicating greater intolerance of uncertainty (α = 0.92).

Meaning in Life

Meaning in life was assessed using the Meaning in Life Questionnaire—MLQ (Steger et al., 2006), which evaluates individuals’ perceptions of meaning in their lives through two dimensions: presence of meaning (e.g., “I understand the meaning of my life”) and search for meaning (e.g., “I am looking for something that makes my life feel meaningful”; α = 0.87). Participants rated their agreement with 10 statements (five for each dimension) on a 7-point Likert scale ranging from 1 (*strongly disagree*) to 7 (*strongly agree*). In the present study, higher scores in the “presence of meaning” subscale reflect a stronger sense of life meaning (α = 0.89).

#### 2.2.4. The Outcome Variables

Parental Stress

Parental stress was assessed using the Parental Stress Scale—PSS (Berry & Jones, 1995), which measures perceptions and feelings regarding parenting experience. The scale captures both the positive aspects of parenthood (e.g., emotional benefits, personal development) and the negative aspects (e.g., demands on resources, feelings of stress). Participants rated their agreement with 18 statements (e.g., “I feel overwhelmed by the responsibilities of being a parent” and “The major source of stress in my life is my children”) on a 7-point Likert scale, ranging from 1 (*strongly disagree*) to 7 (*strongly agree*). Items reflecting positive aspects of parenthood on the Parental Stress Scale (PSS) were reverse coded prior to analysis to ensure consistent scoring across all items. (α = 0.87).

Mental Health

Mental health was measured using the Mental Health Inventory—MHI-5 (Berwick et al., 1991). This scale assesses mental health status through a five-item screening test that evaluates feelings of anxiety, depression, positive affect, and behavioral/emotional control. Participants were asked to indicate the extent to which they had felt the following way during the last few weeks since the war broke out, using a scale from 1 (*very little*) to 7 (*very much*): “nervous and tense” (anxiety), “calm and peaceful” (positive affect), “discouraged and depressed” (depression), “happy and joyful” (positive affect), and “so down in the dumps that nothing could cheer you up” (behavioral/emotional control). Items were coded so that higher scores reflect greater mental health (α = 0.87).

### 2.3. Ethics Statement

Participation in this study was voluntary, and participants were aware that they could withdraw from the study at any time. All participants signed and provided online informed consent. No social security numbers or other identifying data were collected, nor were any invasive examinations conducted. This project was conducted with the approval of the Ethics Committee (IRB) of Hadassah Academic College.

### 2.4. Statistical Analysis

First, descriptive statistics (frequencies distributions or means and standard deviations) related to background and sociodemographic information for the participants were performed as well as examining whether there are differences between the two sub-samples to identify potential covariates.

Then, independent sample t-tests were used to compare the responses of parents of children without special needs with parents of children with special needs on the study main variables: adult attachment patterns (anxiety and avoidance), parental stress, mental health, sense of competence, perceived social support, intolerance of uncertainty, and meaning in life.

Next, Pearson bivariate correlation tests were performed on the sample as a whole and separately for the reports of parents of children with special needs and those of children without special needs to test the associations among adult attachment patterns (anxiety and avoidance), parental stress, mental health, sense of competence, perceived social support, intolerance of uncertainty, and meaning in life. Data were evaluated using the statistical program SPSS version 26 (SPSS Inc., Chicago, IL, USA).

Finally, we analyzed the *total effects* models of attachment patterns on parental stress and mental health while controlling for the shared variance among predictors and the shared variance among the outcomes. Subsequently, we analyzed the *direct-indirect* effects models (*mediational*). These models were conducted using Path Analyses with AMOS (Version 29, (Arbuckle, 2023)) using the maximum-likelihood method. Models were performed first for the sample as a whole, and then a multigroup analysis was conducted to estimate the simultaneous models for the sample of parents of children with special needs and the sample of parents of children without special needs. In this stage of analysis, path coefficients were estimated using 5000 bootstrap samples. All bootstrap samples (100%) converged. Results show that the 95% confidence intervals and the percentile boot-strap confidence intervals for the estimated parameters and indirect effects support the conclusion that the indirect effects are significantly different from zero. The results indicate that the procedure provided a stable estimate of the distributions. We performed all statistical tests using two-tailed tests of significance and confidence intervals based on the level of *p* < 0.05.

## 3. Results

### 3.1. Background and Sociodemographic Variables

Table 1 provides descriptive statistics for participant background and sociodemographic characteristics. A comparison of parents of children with and without special needs revealed no statistically significant differences in the distribution of most demographic variables. Specifically, no significant group differences were found for measures of political affiliation or cumulative exposure to the events of October 7th. However, although in both sub-samples the majority were married, a statistically significant difference was observed in marital status, with parents of children with special needs showing a significantly higher proportion of divorced individuals. Furthermore, this group also exhibited a statistically significant higher mean age and number of children.

To control for potential confounding effects of age, marital status, and number of children, these variables were included as covariates in the subsequent path analyses. Preliminary analyses indicated that controlling for parental gender, age, and number of children did not alter the main study findings. Therefore, these variables were not included in subsequent analyses to maintain clarity and conciseness.

### 3.2. Comparing Responses of Parents of Children with (n = 540) and Without (n = 1557) Special Needs on the Study Main Variables

Independent samples t-tests were employed to assess group differences between parents of children with and without special needs on seven key variables: adult attachment anxiety, adult attachment avoidance, parental stress, mental health, parental sense of competence, perceived social support, and intolerance of uncertainty. Additionally, an independent samples t-test was conducted to compare the groups’ levels of perceived meaning in life. As shown in Table 2, statistically significant differences were observed between the groups across six of the seven variables. Specifically, parents of children with special needs exhibited significantly higher levels of attachment anxiety, attachment avoidance, parental stress, and intolerance of uncertainty. Conversely, this group reported significantly lower levels of parental competence, perceived social support, and mental health. No statistically significant difference in perceived meaning in life was observed between the two groups.

The correlation coefficients can be found in Table 3.

Bivariate correlation analyses were conducted to examine the relationships among the study variables, both for the entire sample (*N* = 2097) and separately for the two subgroups: parents of children with special needs (*n* = 540) and parents of children without special needs (*n* = 1557). The results (Table 3) revealed consistent patterns across both the full sample and the subgroups. Specifically, significant negative correlations were observed between insecure attachment styles (both anxious and avoidant) and measures of parental competence, perceived social support, meaning in life, and mental health. Conversely, significant positive correlations were observed between insecure attachment and parental stress and intolerance of uncertainty. Furthermore, higher levels of parental competence, social support, and meaning in life were significantly associated with lower levels of parental stress and higher levels of mental health, while higher intolerance of uncertainty was associated with higher levels of parental stress and lower levels of mental health.

### 3.3. Multivariate Analyses

#### 3.3.1. The Total Effect

To assess the *total effects* of adult attachment patterns on parental stress and mental health, the path analyses, controlling for the shared variance (intercorrelations) among the predictor variables (attachment anxiety and avoidance) and the outcome variables (parental stress and mental health), were conducted separately for the entire sample and for the two subgroups (parents of children with and without special needs).

The results (Table 4) indicated that in both the entire sample and the subgroups, attachment anxiety was significantly associated with higher levels of parental stress and lower levels of mental health. Conversely, attachment avoidance was significantly associated with lower levels of parental stress and higher levels of mental health.

#### 3.3.2. The Direct Indirect Effect

Path analyses were conducted to assess both the *direct* and *indirect effects* of adult attachment patterns (anxiety and avoidance) on parental stress and mental health. These analyses incorporated a mediational model, controlling for shared variance (intercorrelations) among the predictor variables (attachment anxiety and avoidance), the mediator variables (parental sense of competence, perceived social support, intolerance of uncertainty, and meaning in life), and the outcome variables (parental stress and mental health). Three separate path analyses were performed: one for the full sample and one for each of the two subgroups (parents of children with and without special needs). The results of these analyses are presented in Table 5, Table 6 and Table 7.

**Figure 2:**
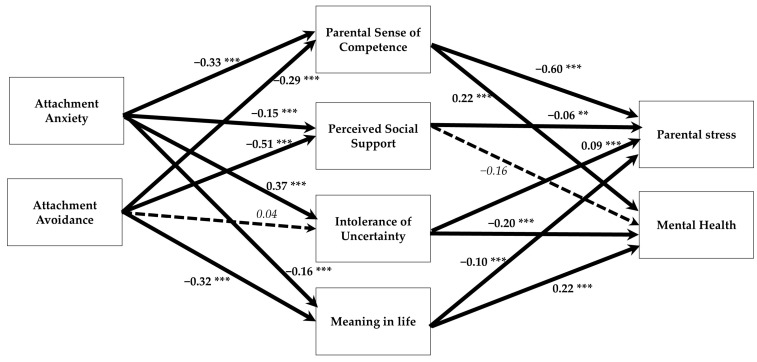
The mediation model for the entire sample. ** *p* < 0.01; *** *p* < 0.001. A dashed arrow indicates a statistically non-significant path. Bold arrows are significant paths, and dashed arrows are nonsignificant paths.

As shown in Table 5, controlling for mediators (parental sense of competence, perceived social support, uncertainty tolerance, meaning in life), the significant total effects of attachment anxiety on parental stress and mental health decreased, while the effects of attachment avoidance became non-significant. The mediated indirect associations of both attachment patterns with high levels of parental stress and low levels of mental health were found to be significant (Figure 2). Intolerance of uncertainty did not mediate the effect of attachment avoidance; nor did perceived social support mediate the effects of either attachment pattern on mental health. This model explained 26%, 34%, 15%, 16%, 61%, and 23% of the variance in parental sense of competence, perceived social support, intolerance of uncertainty, meaning in life, parental stress, and mental health, respectively.

**Figure 3:**
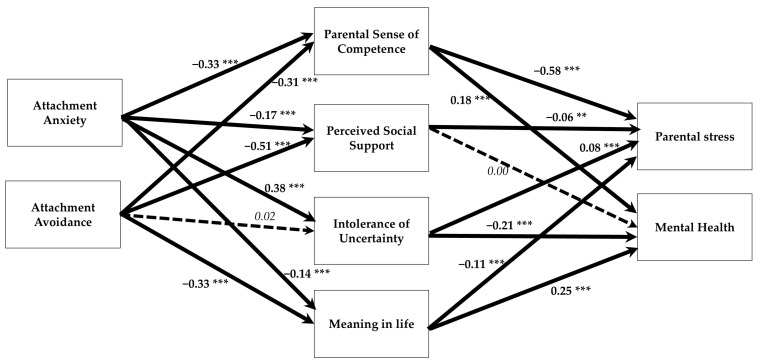
The mediation model for the sample of the parents with children without special needs. ** *p* < 0.01; *** *p* < 0.001. A dashed arrow indicates a statistically non-significant path. Bold arrows are significant paths, and dashed arrows are nonsignificant paths.

Consistent with the full sample (Table 5), analyses for parents of typically developing children (Table 6) showed that the significant total effects of attachment anxiety decreased or became non-significant, and the significant total effects of attachment avoidance became non-significant, when controlling for the mediators. The mediated indirect associations of both attachment styles with high levels of parental stress and low levels of mental health were found to be significant (Figure 3). However, neither intolerance of uncertainty nor perceived social support mediated the effects of either attachment pattern on mental health. This model explained 28%, 35%, 15%, 17%, 61%, and 23% of the variance in parental sense of competence, social support, intolerance of uncertainty, meaning in life, parental stress, and mental health, respectively.

**Figure 4:**
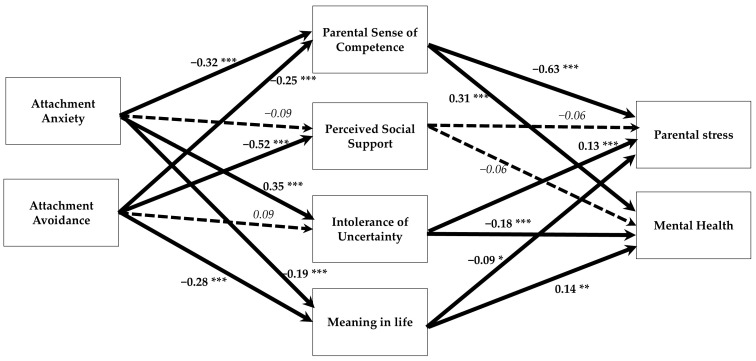
The mediation model for the sample of the parents of children with special needs. * *p* < 0.05; ** *p* < 0.01; *** *p* < 0.001. A dashed arrow indicates a statistically non-significant path. Bold arrows are significant paths, and dashed arrows are nonsignificant paths.

For parents of children with special needs (Table 7), the pattern of mediation effects was similar to but stronger than that observed in the full sample and the subgroup of parents of typically developing children (Table 5 and Table 6). Controlling for the mediators, all total effects of attachment anxiety and avoidance on parental stress and mental health became non-significant. The mediated indirect associations of both attachment patterns with high levels of parental stress and low levels of mental health were found to be significant (Figure 4). Neither intolerance of uncertainty nor perceived social support mediated these indirect effects. This model explained 21%, 30%, 15%, 14%, 62%, and 23% of the variance in parental sense of competence, perceived social support, intolerance of uncertainty, meaning in life, parental stress, and mental health, respectively.

### 3.4. Summary of Findings

This study revealed significant differences in several key variables between parents of children with special needs and those with typically developing children. Parents of children with special needs exhibited significantly higher levels of attachment anxiety, attachment avoidance, parental stress, and intolerance of uncertainty, along with significantly lower levels of parental competence, perceived social support, and mental health. Meaning in life did not differ significantly between groups.

Correlational analyses showed that insecure attachment (both anxiety and avoidant) negatively correlated with parental sense of competence, perceived social support, meaning in life, and mental health while positively correlating with parental stress and intolerance of uncertainty. Conversely, higher parental sense of competence, perceived social support, and meaning in life, combined with lower intolerance of uncertainty, predicted lower parental stress and better mental health.

Mediation analyses demonstrated that insecure attachment significantly predicted increased parental stress and reduced mental health. These effects were mediated by lower parental competence and diminished meaning in life. Intolerance of uncertainty further mediated the effect of attachment anxiety on mental health. However, the mediating role of perceived social support on parental stress, observed in the overall sample and among parents of typically developing children, was absent among parents of children with special needs. These mediation models explained a substantial proportion of the variance in the outcome variables, although the specific variance explained differed somewhat across the subgroups.

## 4. Discussion

This study investigated the impact of the 7 October 2023 attacks in Israel on the mental health and parental stress levels of Israeli parents raising children with and without special needs. The findings reveal significant disparities between these two groups, extending existing research on the challenges faced by parents of children with special needs and demonstrating the profound and compounding effects of external stressful events on parental stress and parents’ overall mental well-being.

The results support Hypothesis 1, showing that parents of children with special needs reported significantly higher levels of parental stress while exhibiting significantly lower levels of parental sense of competence, perceived social support, and overall mental health compared to parents of typically developing children. This aligns with the extensive body of literature documenting the substantial psychological burden on parents of children with special needs, including increased levels of anxiety, depression, and feelings of isolation and burden (Faramarzi, 2017; Wahab & Ramli, 2022). The chronic challenges of specialized care, financial strain, and navigating complex systems contribute to this increased vulnerability and diminished mental health (Isa et al., 2016; Padhyegurjar et al., 2018). The lack of a significant difference in reported meaning in life between the two groups is noteworthy. This unexpected finding (which was not hypothesized) might suggest that while the *sources* of stress differ significantly, the overall sense of purpose and meaning in life may not be related to having or not having children with special needs. However, this could also reflect limitations of the cross-sectional design or the specific measure of meaning employed. Future qualitative research incorporating in-depth interviews could provide valuable insights into this nuanced aspect of the study, particularly in understanding individual variations in experiences of meaning among parents. Our results clarify some of the inconsistencies found in previous research regarding the link between attachment patterns and parental stress by demonstrating that whilst both attachment anxiety and attachment avoidance were associated with parental stress, when controlling for the mediators, the effect of attachment anxiety on parental stress decreased, while the effect of attachment avoidance on parental stress became non-significant. The mediating role of parental sense of competence, perceived social support, intolerance of uncertainty, and meaning in life provide a nuanced understanding of the multiple factors that can influence how insecure attachment patterns impact parental stress and mental health outcomes differently. Overall, these findings underscore the increased vulnerability of parents of children with special needs to both acute and chronic stressors and their detrimental impact on mental well-being, highlighting the need for a deeper understanding of the unique stressors they face.

The findings of the current study regarding the mediating role of meaning in life in the association between attachment patterns, parental stress, and mental health are consistent with previous research—e.g., (Bodner et al., 2014; Mikulincer & Shaver, 2013) that demonstrated an association between attachment patterns and meaning in life. Specifically, secure attachment supports the experience of meaning in life due to several fundamental characteristics. First, the secure base experienced by individuals with secure attachment allows them to explore the world with curiosity and confidence, enabling them to find purpose and meaning in the various domains of their lives. Additionally, secure attachment predicts stable and meaningful interpersonal relationships, which provide a sense of belonging that serves as a core feature of meaning in life.

In contrast, individuals with insecure attachment are more likely to experience feelings of meaninglessness in life, specifically, those with high levels of attachment anxiety, who tend to struggle with low self-efficacy and low self-worth (Shaver & Mikulincer, 2007), making it difficult for them to evaluate their actions across different life domains as meaningful. Their difficulty in maintaining close and valued relationships over time also hinders their ability to feel a sense of belonging, which is a key predictor of meaning in life.

Similarly, individuals with high levels of attachment avoidance tend to adopt a negative model of others, accompanied by feelings of emotional distance and alienation, which limits their ability to derive meaning from interpersonal relationships. More broadly, their tendency to view the world with skepticism, and sometimes even cynicism, makes it difficult for them to perceive value and meaning in their actions beyond their instrumental purposes.

Furthermore, consistent with Hypothesis 2, insecure attachment (both anxiety and avoidant) was significantly correlated with increased parental stress and diminished mental health. This association was mediated by decreased parental sense of competence and a reduced sense of meaning in life, supporting Hypothesis 3. This suggests that individuals with insecure attachment patterns are less well-equipped to effectively manage the heightened stress associated with parenting, leading to negative consequences for both their mental health and their parental roles. This aligns with research demonstrating the link between insecure attachment and maladaptive coping strategies, exacerbating stress and negatively impacting mental health—e.g., (Mikulincer & Shaver, 2023; Shaver & Mikulincer, 2007). Additionally, intolerance of uncertainty significantly mediated the association between attachment anxiety and poorer mental health (supporting H3), consistent with research showing that individuals with high intolerance of uncertainty experience increased anxiety and struggle to cope effectively with ambiguous situations (e.g., Carleton et al., 2012; Bomyea et al., 2015). The heightened uncertainty inherent in raising a child with special needs, exacerbated by the acute stress of a major stressful event like the October 7th attacks, likely contributed significantly to this effect. As discussed in the introduction, prior research in Israel highlighted the impact of socio-emotional factors on psychological responses to war and terror (Ben Avraham et al., 2022; Besser & Neria, 2010, 2012; Besser et al., 2009; Besser & Priel, 2010; Weinberg et al., 2016), directly supporting the findings of this study.

These findings align with prior research—e.g., (Sternheim et al., 2017; Wright et al., 2017) and underscore fundamental principles of attachment theory, particularly the concept of a “secure base” that allows individuals to explore their environment with confidence, knowing that they have a “safe haven” to return to in times of distress (Ainsworth et al., 1978/2015). While secure attachment fosters resilience in unfamiliar situations, individuals with high levels of attachment anxiety often perceive such situations as overly threatening and difficult to manage. Empirical evidence supports this, as Zdebik et al. (2018) found that children with an anxious-ambivalent attachment style at age six exhibited heightened levels of IU by age 21 (Zdebik et al., 2018), and Sanchez et al. (2016) identified a link between the IU levels of mothers and their children, suggesting intergenerational transmission of anxiety (Sanchez et al., 2016). These findings highlight the importance of targeted interventions to support parents, especially those raising children with special needs, in managing high-stress environments, such as those experienced in Israel following October 7th, to mitigate both immediate and long-term psychological impacts on families.

However, the role of perceived social support presented a more nuanced and complex picture, offering only partial support for H3 and contradicting H4. While perceived social support significantly mediated the relationship between attachment patterns and parental outcomes in the overall sample and among parents of typically developing children, this effect was notably absent among parents of children with special needs. This aligns with research showing that pre-existing social isolation and limited societal support are frequently experienced by parents of children with special needs (Chen et al., 2017), significantly constraining their ability to leverage available social support resources in the aftermath of the October 7th attacks. Furthermore, the unique cultural context and societal perceptions surrounding parenting children with special needs likely exacerbate feelings of isolation and hinder access to supportive networks. Understanding these barriers is essential for designing interventions that genuinely enhance support networks for these families. The pre-existing feelings of isolation, frustration, and stigma experienced by this population (Leitch et al., 2019) likely exacerbated this effect, diminishing the effectiveness of available support in mitigating stress and improving mental health. The absence of mediation in this subgroup might not reflect simply a lack of resources but might rather reflect a pre-existing inability to effectively access and utilize those resources due to complex social and psychological factors. As such, intervention strategies must be specifically tailored to account for these barriers in order to be effective. This finding highlights an important area for intervention, as discussed in the introduction, where bolstering social support networks and directly addressing feelings of isolation and marginalization are crucial for improving the well-being of parents of children with special needs.

The findings regarding the mediating role of perceived social support in the association between attachment patterns and parental stress are consistent with previous research. For example, McMahon et al. (2020) found that perceived social support mediates the association between attachment anxiety and cardiovascular stress reactivity (McMahon et al., 2020). Similarly, Chen et al. (2017) demonstrated that perceived social support mediates the association between attachment to parents and life satisfaction among adolescents (Chen et al., 2017). Sirois et al. (2016) showed that perceived social support mediates the link between attachment anxiety and well-being in a longitudinal study that followed emerging adults over several weeks (Sirois et al., 2016). Additionally, future studies should explore how specific types of social support, such as emotional, informational, and tangible support, differentially influence parental stress outcomes, particularly for those raising children with special needs. In a study conducted in Israel during the first lockdown imposed in March 2020 following the outbreak of the COVID-19 pandemic, perceived social support mediated the association between attachment anxiety and lower levels of positive affect (Alfasi, 2023).

The study has several limitations. The cross-sectional design prevents causal inferences; longitudinal research is needed to determine the temporal relationships between these variables. The reliance on self-report measures introduces potential response bias. The convenience sample, while offering strong ecological validity by focusing on parents’ subjective experiences, may not fully represent the diverse population of Israeli parents, potentially limiting the generalizability of these findings. Specifically, future research should aim to diversify its sample to include parents from various socio-economic and cultural backgrounds to ensure the robustness of the findings. Future research should investigate specific mechanisms through which attachment patterns influence coping strategies among parents of children with special needs. Additionally, longitudinal studies should seek to understand how perceptions of social support evolve over time in response to traumatic events. It is also essential for future studies to address potential alternative explanations for the findings to enhance the depth of analysis. Future research should explicitly address these limitations through the application of longitudinal designs, the inclusion of objective measures of parental stress and mental health, and the recruitment of more diverse and representative samples. It is important to acknowledge the heterogeneity of special needs; children within this group experience diverse challenges, ranging in severity and type. Further studies should include larger groups of diverse special needs and severity measures and should account for this variability. Finally, while a model positing direct mediation of the relationship between parental sense of competence and outcomes by attachment patterns is plausible, our mediational model (Figure 1) is better supported by established attachment theory and research. Attachment pattern, as a *pre-existing personality predisposition*, is more accurately viewed as influencing the mediating variables—parental sense of competence, perceived social support, intolerance of uncertainty, and meaning in life—which in turn, impact parental stress and mental well-being.

Despite these limitations, the findings have important implications for practice and policy. This study’s findings emphasize the need to develop interventions that address the specific stressors faced by parents of children with special needs, ensuring that these interventions are culturally sensitive and trauma informed. Additionally, future policies should prioritize the establishment of robust support networks that cater specifically to this vulnerable population. Calling for a systemic approach, interventions should not only focus on immediate support but also aim to empower parents by providing strategies to improve their resilience and coping mechanisms. Culturally sensitive, trauma-informed interventions and support systems are urgently needed to enhance parental competence, strengthen social support networks, and implement effective stress management strategies tailored to the unique needs of parents raising children with special needs. These interventions must explicitly address both pre-existing vulnerabilities (related to parenting children with special needs) and acute stressors (such as the October 7th attacks) to promote parental well-being and support healthy child development. Future research should rigorously evaluate the efficacy of various interventions aimed at improving parental resilience and mental health within this vulnerable population. Such efforts will be important in ensuring that support systems are effective and responsive to the unique challenges faced by these families.

## 5. Conclusions

This study confirms that the unique challenges of raising children with special needs significantly impact parental stress and overall mental health and that these effects are exacerbated by external stressful events. Our findings underscore the important need for culturally sensitive, trauma-informed interventions and support systems tailored to the specific needs of parents of children with special needs, promoting both parental resilience and, ultimately, the well-being of their children. Future research should focus on longitudinal studies, mixed-methods designs incorporating qualitative data, and rigorous evaluations of intervention efficacy.

## Figures and Tables

**Table 1:** Sociodemographic and background information.

Variables	Total Sample (*N* = 2097)	Parents of Children with Special Needs (*n* = 540)	Parents of Children Without Special Needs (*n* = 1557)	Statistic
**Sex**				χ^2^_[df=1]_ = 0.22, *ns*
*Men*	48%	48.9%	47.7%	
*Women*	52%	51.1%	52.3%	
**Age**	37.96 (7.54)	40.66 (6.12)	37.03 (7.76)	t_[df=2095]_ = −9.87, *p* < 0.001
**Number of children**	3.54 (1.29)	4.02 (1.34)	3.37 (1.24)	t_[df=2095]_ = −10.27, *p* < 0.001
**Right-wing Political Stance**	5.22 (1.47)	5.17 (1.48)	5.24 (1.47)	t_[df=2095]_ = 0.84, *ns*
**Cumulative Exposure**	2.21 (1.09)	2.26 (1.05)	2.19 (1.10)	t_[df=2095]_ = −1.34, *ns*
**Employment Status**				
*Full time*	75.3%	76.7%	74.8%	χ^2^_[df=9]_ = 9.26, *ns*
*Part time*	12.6%	13.0%	12.5%	
*On leave*	1.0%	0.7%	1.1%	
*Temporarily laid off*	0.5%	0.2%	0.6%	
*Unemployed and looking for work*	2.2%	2.8%	2.1%	
*Unemployed health-related reason*	0.8%	0.7%	0.8%	
*Going to school*	5.3%	3.7%	5.8%	
*Home maker*	1.5%	1.1%	1.7%	
*Retired*	0.0%	0.0%	0.1%	
*Disability payments*	0.7%	1.1%	0.6%	
**Religiosity**				χ^2^_[df=4]_ = 5.15, *ns*
*Atheist*	5.6%	5.0%	5.8%	
*Secular*	41.4%	39.8%	42.0%	
*Traditional*	19.0%	21.9%	18.0%	
*Religious*	15.1%	15.9%	14.8%	
*Ultra-Orthodox*	18.9%	17.4%	19.4%	
**Marital Status**				χ^2^_[df=7]_ = 25.11, *p* < 0.001
*Single*	2.6%	2.8%	2.6%	
*Casually dating*	0.0%	0.0%	0.1%	
*Seriously dating*	0.6%	0.2%	0.8%	
*Cohabiting*	3.6%	2.8%	3.9%	
*Married*	85.9%	82.8%	87.0%	
*Separated*	0.8%	1.3%	0.6%	
*Divorced*	6.3%	10.2%	5.0%	
*Widowed*	0.1%	0.0%	0.1%	
**Education**				χ^2^_[df=4]_ = 2.42, *ns*
*Elementary education*	15.7%	14.4%	16.1%	
*S* *econdary education*	20.0%	20.6%	19.8%	
*Non-academic post-secondary education*	40.7%	40.7%	40.7%	
*Bachelor’s degree*	21.5%	21.5%	21.5%	
*Master’s degree or higher*	2.1%	2.8%	1.9%	
**Monthly gross household income**				χ^2^_[df=4]_ = 2.64, *ns*
*Below 5000 NIS*	6.2%	6.5%	6.1%	
*5000–10,000 NIS*	27.7%	25.6%	28.5%	
*10,000–15,000* *NIS*	37.1%	36.7%	37.2%	
*15,000–20,000 NIS*	19.8%	21.5%	19.2%	
*Above 20,000 NIS*	9.3%	9.8%	9.1%	
**Place of Residence**				χ^2^_[df=4]_ = 1.59, *ns*
*Northen*	22.3%	23.5%	21.9%	
*Central*	39.3%	38.0%	39.8%	
*Coastal Plain*	9.8%	10.6%	9.5%	
*Jerusalem Region*	16.0%	15.2%	16.3%	
*Southern*	12.6%	12.8%	12.5%	

*ns* = non-significant; *NIS* = New Israeli Shekel (currency).

**Table 2:** Comparisons of parents of children with and without special needs.

Variables	Parents of Children with Special Needs (*n* = 540)		Parents of Children Without Special Needs (*n* = 1557)		t_[df=2095]_
	*M*	*SD*	*M*	*SD*	
1. Attachment Anxiety	3.06	1.30	2.91	1.20	−2.47 *
2. Attachment Avoidance	2.65	1.19	2.46	1.12	−3.35 ***
3. Parental Sense of Competence	4.64	0.86	4.84	0.81	4.95 ***
4. Perceived Social Support	5.34	1.32	5.50	1.22	2.62 **
5. Intolerance of Uncertainty	4.19	1.23	4.07	1.21	−2.04 *
6. Meaning in Life	5.13	1.41	5.16	1.33	0.49
7. Parental Stress	3.04	0.86	2.83	0.79	−5.29 ***
8. Mental Health	3.64	1.36	3.86	1.34	3.25 ***

* *p* < 0.05; ** *p* < 0.01; *** *p* < 0.0013.2. Univariate analyses.

**Table 3:** Intercorrelations.

The Entire Sample (*N* = 2097)	1	2	3	4	5	6	7	8
1. Attachment Anxiety	*-*							
2. Attachment Avoidance	0.35 ***	-						
3. Parental Sense of Competence	−0.43 ***	−0.41 ***	-					
4. Perceived Social Support	−0.33 ***	−0.56 ***	0.41 ***	-				
5. Intolerance of Uncertainty	0.39 ***	0.17 ***	−0.38 ***	−0.17 ***	-			
6. Meaning in Life	−0.27 ***	−0.37 ***	0.48 ***	0.45 ***	−0.18 ***	-		
7. Parental Stress	0.44 ***	0.39 ***	−0.76 ***	−0.41 ***	0.39 ***	−0.47 ***	-	
8. Mental Health	−0.26 ***	−0.17 ***	0.39 ***	0.20 ***	−0.33 ***	0.35 ***	−0.34 ***	-
**For Each Sample**	**1**	**2**	**3**	**4**	**5**	**6**	**7**	**8**
1. Attachment Anxiety	*-*	0.27 ***	−0.39 ***	−0.23 ***	0.38 ***	−0.26 ***	0.38 ***	−0.26 ***
2. Attachment Avoidance	0.38 ***	-	−0.33 ***	−0.54 ***	0.18 ***	−0.33 ***	0.32 ***	−0.15 ***
3. Parental Sense of Competence	−0.44 ***	−0.43 ***	-	0.40 ***	−0.36 ***	0.50 ***	−0.77 ***	0.43 ***
4. Perceived Social Support	−0.36 ***	−0.57 ***	0.41 ***	-	−0.14 ***	0.50 ***	−0.39 ***	0.17 ***
5. Intolerance of Uncertainty	0.39 ***	0.17 ***	−0.39 ***	−0.18 ***	-	−0.17 ***	0.40 ***	−0.32 ***
6. Meaning in Life	−0.27 ***	−0.39 ***	0.48 ***	0.43 ***	−0.18 ***	-	−0.47 ***	0.31 ***
7. Parental Stress	0.46 ***	0.42 ***	−0.75 ***	−0.42 ***	0.38 ***	−0.47 ***	-	−0.39 ***
8. Mental Health	−0.26 ***	−0.17 ***	0.37 ***	0.20 ***	−0.33 ***	0.36 ***	−0.31 ***	-

Values *below* the diagonal are taken from the sample of parents of children without special needs (*n* = 1557), whereas the values *above* the diagonal are taken from the sample of parents of children with special needs (*n* = 540). *** *p* < 0.001.

**Table 4:** Results of the total effects path analyses for attachment patterns.

Total Effect	Estimates			
	Standardized Path Coefficients	SE	*t*	*p*<
*Sample as a whole (N = 2097)*				
Attachment Anxiety → Parental Stress	0.35	0.01	17.37	0.0001
Attachment Anxiety → Mental Health	−0.23	0.03	−10.02	0.0001
Attachment Avoidance → Parental Stress	0.27	0.01	13.62	0.0001
Attachment Avoidance → Mental Health	−0.09	0.03	−4.14	0.0001
*Parent of child without special needs (n = 1557*)				
Attachment Anxiety → Parental Stress	0.36	0.02	15.36	0.0001
Attachment Anxiety → Mental Health	−0.22	0.03	−8.37	0.0001
Attachment Avoidance → Parental Stress	0.28	0.02	12.25	0.0001
Attachment Avoidance → Mental Health	−0.09	0.03	−3.37	0.0001
*Parents of child with special needs (n = 540)*				
Attachment Anxiety → Parental Stress	0.32	0.03	8.05	0.0001
Attachment Anxiety → Mental Health	−0.23	0.05	−5.35	0.0001
Attachment Avoidance → Parental Stress	0.23	0.03	5.77	0.0001
Attachment Avoidance → Mental Health	−0.09	0.05	−2.11	0.03

**Table 5:** Results of the path mediation analyses for attachment patterns (sample as a whole, *N* = 2097).

Effect	Estimates				Bootstrap					
								95% PC Confidence Interval		
	Standardized Path Coefficients	SE	*t*	*p*<	SE	Bias	SE-Bias	Lower Bound	Upper Bound	*p*
***Associations with Mediators***										
Attachment Anxiety → Sense of Competence	−0.33	0.013	−16.43	0.0001	0.014	0.000	0.000	−0.249	−0.193	0.000
Attachment Anxiety → Perceived Social Support	−0.15	0.019	−7.67	0.0001	0.024	0.000	0.000	−0.194	−0.102	0.000
Attachment Anxiety → Intolerance of Uncertainty	0.37	0.021	17.28	0.0001	0.024	0.000	0.000	0.320	0.415	0.000
Attachment Anxiety → Meaning in Life	−0.16	0.024	−7.29	0.0001	0.027	0.000	0.000	−0.224	−0.119	0.000
Attachment Avoidance → Sense of Competence	−0.29	0.015	−14.58	0.0001	0.016	0.000	0.000	−0.244	−0.181	0.000
Attachment Avoidance → Perceived Social Support	−0.51	0.021	−26.94	0.0001	0.026	0.000	0.000	−0.610	−0.510	0.000
Attachment Avoidance → Intolerance of Uncertainty	0.04	0.023	2.006	0.05	0.025	0.001	0.000	−0.003	0.095	0.061
Attachment Avoidance → Meaning in Life	−0.32	0.025	−14.73	0.0001	0.029	−0.001	0.000	−0.430	−0.318	0.000
***Associations with Outcome***										
Attachment Anxiety → Parental Stress (Direct)	0.09	0.011	5.767	0.0001	0.012	0.000	0.000	0.039	0.085	0.000
Attachment Anxiety → Mental Health (Direct)	−0.05	0.025	−2.167	0.03	0.025	0.000	0.000	−0.104	−0.005	0.028
Attachment Avoidance → Parental Stress (Direct)	0.03	0.012	1.866	0.06	0.013	0.000	0.000	−0.003	0.049	0.083
Attachment Avoidance → Mental Health (Direct)	0.04	0.029	1.583	0.11	0.031	0.001	0.000	−0.015	0.107	0.14
Parental Sense of Competence → Parental Stress	−0.60	0.017	−33.946	0.0001	0.019	0.000	0.000	−0.627	−0.553	0.000
Perceived Social Support → Parental Stress	−0.06	0.011	−3.452	0.0001	0.012	0.000	0.000	−0.063	−0.016	0.002
Intolerance of Uncertainty → Parental Stress	0.09	0.010	6.07	0.0001	0.011	0.000	0.000	0.040	0.084	0.000
Meaning in Life → Parental Stress	−0.10	0.010	−6.052	0.0001	0.011	0.000	0.000	−0.081	−0.039	0.000
Parental Sense of Competence → Mental Health	0.22	0.041	8.638	0.001	0.046	0.000	0.001	0.261	0.437	0.000
Perceived Social Support → Mental Health	−0.16	0.027	−0.634	0.526	0.027	0.000	0.000	−0.071	0.037	0.554
Intolerance of Uncertainty → Mental Health	−0.20	0.024	−9.189	0.0001	0.026	0.000	0.000	−0.271	−0.167	0.000
Meaning in Life → Mental Health	0.22	0.023	9.279	0.0001	0.024	0.000	0.000	0.166	0.263	0.000
***Indirect effect***										
Attachment Anxiety → Parental Stress	0.26				0.016			0.147	0.192	0.000
Attachment Anxiety → Mental Health	−0.18				0.015			−0.226	−0.161	0.000
Attachment Avoidance → Parental Stress	0.24				0.017			0.148	0.199	0.000
Attachment Avoidance → Mental Health	−0.13				0.017			−0.195	−0.116	0.000

Based on 5000 bootstrap samples. PC confidence = percentile confidence intervals (95%).

**Table 6:** Results of the path mediation analyses for attachment patterns (parent of child without special needs, *n =* 1557).

Effect	Estimates				Bootstrap					
								95% PC Confidence Interval		
	Standardized Path Coefficients	SE	*t*	*p*<	SE	Bias	SE-Bias	Lower Bound	Upper Bound	*p*
***Associations with Mediators***										
Attachment Anxiety → Sense of Competence	−0.33	0.016	−14.10	0.0001	0.016	0.000	0.000	−0.252	−0.188	0.000
Attachment Anxiety → Perceived Social Support	−0.17	0.022	−7.713	0.0001	0.027	0.000	0.000	−0.226	−0.119	0.000
Attachment Anxiety → Intolerance of Uncertainty	0.38	0.025	15.025	0.0001	0.028	0.000	0.000	0.325	0.439	0.000
Attachment Anxiety → Meaning in Life	−0.14	0.028	−5.699	0.0001	0.030	0.000	0.000	−0.216	−0.098	0.000
Attachment Avoidance → Sense of Competence	−0.31	0.017	−13.17	0.0001	0.018	0.000	0.000	−0.257	−0.186	0.000
Attachment Avoidance → Perceived Social Support	−0.51	0.024	−22.91	0.0001	0.030	0.000	0.000	−0.610	−0.492	0.000
Attachment Avoidance → Intolerance of Uncertainty	0.02	0.027	0.903	0.367	0.030	−0.001	0.000	−0.034	0.083	0.427
Attachment Avoidance → Meaning in Life	−0.33	0.030	−13.21	0.0001	0.033	0.000	0.000	−0.457	−0.329	0.000
***Associations with Outcome***										
Attachment Anxiety → Parental Stress (Direct)	0.11	0.013	5.768	0.0001	0.014	0.000	0.000	0.045	0.100	0.000
Attachment Anxiety → Mental Health (Direct)	−0.05	0.030	−1.86	0.06	0.030	0.001	0.000	−0.113	0.006	0.076
Attachment Avoidance → Parental Stress (Direct)	0.04	0.014	1.90	0.06	0.015	0.000	0.000	−0.002	0.057	0.071
Attachment Avoidance → Mental Health (Direct)	0.05	0.034	1.81	0.07	0.039	0.000	0.001	−0.016	0.137	0.113
Parental Sense of Competence → Parental Stress	−0.58	0.020	−27.83	0.0001	0.022	0.000	0.000	−0.608	−0.520	0.000
Perceived Social Support → Parental Stress	−0.06	0.013	−2.961	0.003	0.014	−0.001	0.000	−0.068	−0.012	0.005
Intolerance of Uncertainty → Parental Stress	0.08	0.012	4.552	0.0001	0.013	0.000	0.000	0.027	0.080	0.000
Meaning in Life → Parental Stress	−0.11	0.011	−5.718	0.0001	0.012	0.000	0.000	−0.088	−0.041	0.000
Parental Sense of Competence → Mental Health	0.18	0.048	6.043	0.0001	0.053	0.000	0.001	0.189	0.399	0.000
Perceived Social Support → Mental Health	0.00	0.032	0.057	0.96	0.033	−0.001	0.000	−0.066	0.066	0.972
Intolerance of Uncertainty → Mental Health	−0.21	0.028	−8.190	0.0001	0.031	0.000	0.000	−0.287	−0.165	0.000
Meaning in Life → Mental Health	0.25	0.027	9.214	0.0001	0.028	0.001	0.000	0.194	0.304	0.000
***Indirect effect***										
Attachment Anxiety → Parental Stress	0.21				0.018			0.137	0.186	0.000
Attachment Anxiety → Mental Health	−0.21				0.018			−0.230	−0.152	0.000
Attachment Avoidance → Parental Stress	0.21				0.020			0.145	0.203	0.000
Attachment Avoidance → Mental Health	−0.18				0.021			−0.219	−0.120	0.000

Based on 5000 bootstrap samples. PC confidence = percentile confidence intervals (95%).

**Table 7:** Results of the path mediation analyses for attachment patterns (parents of child with special needs, *n* = 540).

Effect	Estimates				Bootstrap					
								95% PC Confidence Interval		
	Standardized Path Coefficients	SE	*t*	*p*<	SE	Bias	SE-Bias	Lower Bound	Upper Bound	*p*
***Associations with Mediators***										
Attachment Anxiety → Sense of Competence	−0.32	0.026	−8.140	0.0001	0.029	0.000	0.000	−0.272	−0.156	0.000
Attachment Anxiety → Perceived Social Support	−0.09	0.038	−2.345	0.02	0.050	0.001	0.001	−0.188	0.10	0.076
Attachment Anxiety → Intolerance of Uncertainty	0.35	0.039	8.588	0.0001	0.046	0.001	0.001	0.246	0.428	0.000
Attachment Anxiety → Meaning in Life	−0.19	0.045	−4.490	0.0001	0.053	0.001	0.001	−0.306	−0.097	0.001
Attachment Avoidance → Sense of Competence	−0.25	0.029	−6.163	0.0001	0.031	0.000	0.000	−0.236	−0.115	0.000
Attachment Avoidance → Perceived Social Support	−0.52	0.042	−13.73	0.0001	0.051	0.000	0.001	−0.671	−0.472	0.000
Attachment Avoidance → Intolerance of Uncertainty	0.09	0.043	2.080	0.04	0.047	−0.001	0.001	−0.006	0.177	0.067
Attachment Avoidance → Meaning in Life	−0.28	0.049	−6.837	0.0001	0.059	0.000	0.001	−0.453	−0.221	0.000
***Associations with Outcome***										
Attachment Anxiety → Parental Stress (Direct)	0.052	0.020	1.722	0.085	0.020	0.000	0.000	−0.005	0.075	0.092
Attachment Anxiety → Mental Health (Direct)	−0.05	0.045	−1.099	0.272	0.047	0.000	0.001	−0.143	0.043	0.290
Attachment Avoidance → Parental Stress (Direct)	0.01	0.023	0.383	0.702	0.025	0.001	0.000	−0.039	0.059	0.691
Attachment Avoidance → Mental Health (Direct)	0.01	0.052	0.216	0.829	0.056	0.000	0.001	−0.099	0.119	0.829
Parental Sense of Competence → Parental Stress	−0.63	0.034	−18.714	0.0001	0.036	0.001	0.001	−0.705	−0.566	0.000
Perceived Social Support → Parental Stress	−0.06	0.023	−1.602	0.11	0.023	0.000	0.000	−0.080	0.011	0.129
Intolerance of Uncertainty → Parental Stress	0.13	0.021	4.240	0.0001	0.022	0.000	0.000	0.045	0.132	0.000
Meaning in Life → Parental Stress	−0.09	0.020	−2.600	0.01	0.023	0.000	0.000	−0.099	−0.009	0.017
Parental Sense of Competence → Mental Health	0.31	0.076	6.346	0.0001	0.084	0.000	0.001	0.316	0.651	0.000
Perceived Social Support → Mental Health	−0.06	0.051	−1.159	0.246	0.050	0.000	0.001	−0.160	0.038	0.234
Intolerance of Uncertainty → Mental Health	−0.18	0.047	−4.184	0.0001	0.052	−0.001	0.001	−0.298	−0.095	0.000
Meaning in Life → Mental Health	0.14	0.045	3.023	0.005	0.049	−0.001	0.001	0.042	0.235	0.005
***Indirect effect***										
Attachment Anxiety → Parental Stress	0.27	0.035						0.200	0.337	0.000
Attachment Anxiety → Mental Health	−0.18	0.028						−0.240	−0.130	0.000
Attachment Avoidance → Parental Stress	0.22	0.035						0.150	0.287	0.000
Attachment Avoidance → Mental Health	−0.10	0.030						−0.160	−0.044	0.000

Based on 5000 bootstrap samples. PC confidence = percentile confidence intervals (95%).

## Data Availability

The data presented in this study are available on request from the corresponding authors.
